# Role of myocardial scar on 30day outcome after TAVI

**DOI:** 10.1186/1532-429X-17-S1-P157

**Published:** 2015-02-03

**Authors:** Britta Butzbach, Sebastian Gruenig, Dominic Prinz, Tobias Zeus, Ralf Westenfeld, Florian Boenner, Malte Kelm, Mirja Neizel-Wittke

**Affiliations:** Department of Cardiology, Angiology, Pneumology, University of Duesseldorf, Cardiology, Düsseldorf, Germany

## Background

Transcatheter aortic valve implantation (TAVI) offers a minimal invasive option for treatment of patients with severe aortic stenosis at high risk for conventional surgery. The objective of this study was to investigate whether presence of late gadolinium enhancement (LGE) patterns detected by cardiac magnetic resonance imaging (cMRI) is a risk factor for major adverse cardiac events (MACE) after TAVI within the first 30 days.

## Methods

77 consecutive patients with severe aortic stenosis (aortic valve area <1cm^2^) underwent cMRI on a 1.5 Tesla MR Scanner (Achieva, Philips, The Netherlands) with gadolinium contrast agent (Dotarem, Guerbet, France) before transcatheter aortic valve implantation (TAVI). Measurements of left ventricular (LV) and right ventricular (RV) volumes, ejection fraction (EF), stroke volume (SV) and LGE patterns (no LGE, transmural, subendocardial scar, midwall fibrosis and fibrosis of the right ventricular insertion points) were determined. Clinical characteristics and 30 days outcome were correlated with cMRI measurements. MACE included death, myocardial infarction, stroke and reintervention.

## Results

Mean age of patients was 83 +/-5 years, 66% of the patients were female. 58 patients showed LGE patterns on MRI: 8 patients had midwall fibrosis, 19 patients had infarct-like myocardial LGE(6 subendocardial scars, 13 transmural scars) and 46 patients had fibrosis of the right ventricular insertion points (31 as single pattern, 15 as combined pattern). Patients with infarct like LGE had worse LVEF than patients with no scar (no scar: 66.5 ± 16%, infarct like scar: 41 ±19%, p=0.01).

Patients with midwall fibrosis more often reported syncopal events before admission (p=0.05) and had worse right ventricular function (RVEF<50%) then patients without LGE (p=0.04). 30 day-MACE was low (5%), 2 patients died (asystolia and combination of severe cardial complications and sepsis) and 2 patients suffered a stroke. MACE was significant higher in patients with midwall fibrosis (p=0.05), however not in the other LGE-groups. Left bundle branch block (p=0.5), high degree AV- block (p=0.9), need of permanent pacemaker (p=0.3), cardiac decompensation (p=0.7), TIA (p=0.9) and rehospitalization (p=0.4) did not show significant differences between the groups.

## Conclusions

Different late gadolinium enhancement patterns exist in patients with aortic stenosis. The presence of LGE of any cause does not appear to predict MACE within the first 30 days. However, MACE was significantly higher in patients with midwall fibrosis. Studies with more patients have to be performed to investigate whether midwall fibrosis in patients scheduled for TAVI is a risk factor for this patient group.

## Funding

Funded by the University of Duesseldorf.

**Figure 1:**
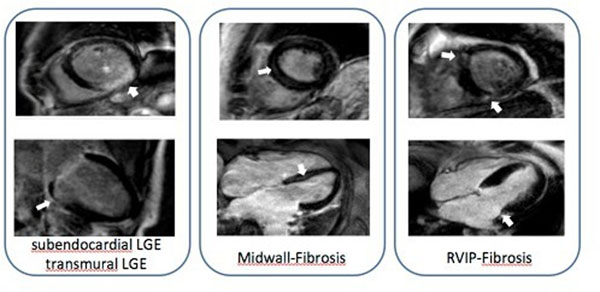
Figure 1

